# Integrated 13C-DNA Stable Isotope Probing and Metagenomics Approaches to Identify Bisphenol A Assimilating Microorganisms and Metabolic Pathways in Biofilms

**DOI:** 10.3390/toxics14010080

**Published:** 2026-01-15

**Authors:** Di Wang, Jiayue Sun, Yunian Zhang, Lingjue Yuan, Xia Xu, Yingang Xue, Haohao Sun

**Affiliations:** 1 School of Environmental Science and Engineering, Changzhou University, Changzhou 213164, China; wangdidi0625@163.com (D.W.); s23030857049@smail.cczu.edu.cn (J.S.); zhangyunian111@163.com (Y.Z.); 15352171818@163.com (L.Y.); 2 College of Urban Construction, Changzhou University, Changzhou 213164, China; xuxia@cczu.edu.cn

**Keywords:** biofilm, bisphenol A, DNA-SIP, assimilating bacteria, metabolic pathway

## Abstract

Bisphenol A (BPA) is a persistent environmental contaminant requiring effective removal strategies. Biofilms offer advantages over conventional activated sludge for refractory compound degradation, yet the specific microorganisms and mechanisms driving BPA removal in biofilms remain poorly understood. This study employed an integrated approach, combining ^13^C-DNA stable isotope probing (SIP) and metagenomics to identify BPA-assimilating microorganisms and elucidate their metabolic pathways in biofilms. Two moving bed biofilm reactors (MBBRs) were operated at contrasting BPA concentrations (500 μg/L and 10 mg/L) to enrich distinct microbial communities. Using DNA-SIP, we revealed differences in assimilating bacteria across diverse concentrations of BPA-enriched biofilms. Simultaneously, we reconstructed the genomes of these assimilating bacteria, dissecting the functional genes essential to the degradation process and identifying significant gene variations among different assimilating bacteria. By integrating these gene functions, we constructed the BPA metabolic pathway, which surprisingly comprised genes from various assimilating bacteria. This research significantly advances our understanding of BPA-assimilating bacteria within biofilms and provides valuable insights for refining biofilm technologies aimed at BPA removal from wastewater.

## 1. Introduction

Bisphenol A (BPA), an anthropogenic compound, was first synthesized in 1890 and has been in commercial use since the 1950s [1]. Its widespread applications include the production of polycarbonates for electrical and electronic equipment, as well as bottles and beverage containers [2]. Despite its gradual elimination from numerous consumer products and industrial formulations, an annual release of over one million pounds of BPA into the environment persists [3]. Wastewater treatment plants (WWTPs) serve as primary recipients of BPA, exhibiting removal efficiencies that vary greatly from 10% to over 99%. Consequently, they are identified as substantial contributors to BPA discharge into the natural environment [4]. The continuous release from WWTPs results in BPA presence within aquatic environments, where its concentrations range from ng/L to μg/L [5,6]. The potential toxic impact of BPA on aquatic organisms is a subject of widespread concern [7]. Concrete evidence of BPA-induced endocrine disruption has emerged across numerous aquatic organisms, encompassing fish, amphibians, and invertebrates [8]. Consequently, the development of effective treatment technologies to remove varying concentrations of BPA from wastewater has become paramount.

In recent years, biological methods have garnered attention for their potential to remove BPA from wastewater, primarily due to their cost-effectiveness and minimal secondary contamination. Among these methods, both activated sludge and biofilm have emerged as popular choices for BPA removal. However, biofilm offers distinct advantages, such as enhanced stability and superior removal efficiency, when compared to conventional activated sludge processes [9]. Bacteria assume a central role in driving BPA removal within biological treatment procedures. An array of assimilating bacteria has been successfully isolated from activated sludge and biofilm environments. For instance, BPA-assimilating bacteria like *Lactococcus lactis* subsp. lactis 712, *Sphingomonas* sp., and *Bacillus* sp. GZB have been extracted from activated sludge [10,11,12], while *Pseudomonas aeruginosa* has been identified within biofilms as a capable BPA degrader [13]. Furthermore, within the realm of activated sludge, select studies have employed DNA stable isotope probing (DNA-SIP) technology to delve into the intricate composition of complex functional bacteria engaged in degradation [14,15]. Beyond investigations into functional bacteria, meticulous analyses of the metabolic pathways employed by BPA-assimilating bacteria within activated sludge have also been conducted. Notably, Yu, Yi, Li, Guo, Peng, Wang, Wu, Alvarez-Cohen, and Zhang [10] have undertaken an intricate dissection of the BPA metabolic pathways within *Sphingomonas*, *Pseudomonas*, and *Pusillimonas*. Regrettably, such research remains relatively scarce when it comes to biofilm applications. This gap in understanding the assimilating bacterial community and metabolic function considerably hampers the advancement of biofilm-based processes tailored for BPA removal. Addressing this knowledge deficit becomes pivotal for realizing the full potential of biofilm technologies in efficiently tackling BPA removal.

In the intricate task of identifying functional bacteria engaged in organic matter degradation within complex environments, DNA-SIP emerges as a notably effective technology [16,17]. This approach involves the initial labeling of substrates of interest with stable isotopes (such as 13C or 15N isotopes). Microorganisms utilizing these isotopically labeled substrates then integrate the stable isotopes into their DNA. The ensuing DNA can be separated from that of other bacteria via density gradient centrifugation [18,19]. This method of analyzing bacterial cell composition furnishes detailed insights into the microorganisms utilizing the labeled substrate. DNA-SIP has found utility in the examination of various organic matter-degrading microorganisms in activated sludge [20,21], encompassing even trace organic compounds like BPA [14,15]. Leveraging the identification of degrading bacteria as a foundation, the BPA degradation mechanisms employed by these bacteria can be subjected to thorough analysis. This involves the identification of functional genes and the construction of metabolic pathways through the genomes of these degrading bacteria [10]. However, the utilization of DNA-SIP for the analysis of organic matter-assimilating bacteria within biofilms has been relatively limited. Furthermore, studies pertaining to the associated metabolic pathways are even scarcer. Addressing these gaps in the research holds the potential to significantly enhance our understanding of the intricate microbial ecology within biofilms. Such insights may prove invaluable in the development of biofilm-based processes, ultimately enriching our arsenal of tools for addressing complex environmental challenges.

In this study, a combination of DNA-SIP and metagenomics was harnessed to meticulously delve into the intricate mechanisms of BPA biodegradation and assimilation within biofilms. This study presents several significant advances in understanding BPA biodegradation in biofilms. By applying DNA-SIP, we identified biofilm-specific BPA-assimilating bacteria under different concentration regimes, directly addressing a key gap in knowledge regarding functional degraders unique to biofilm ecosystems. Furthermore, we integrated functional identification with genomic analysis by reconstructing the genomes of these assimilators and mapping their degradation-related genes—establishing, for the first time, a direct link between biofilm-enriched degraders and their metabolic potential. The reconstruction of BPA metabolic pathways further elucidated how multiple bacterial species collaborate within biofilm consortia to achieve complete mineralization, offering novel insights into cooperative degradation mechanisms. Lastly, comparative analysis between biofilm and activated sludge systems underscored how biofilm-specific microbial architectures support distinct degradation networks, providing a mechanistic basis for the enhanced performance of biofilm-based BPA removal. Collectively, this integrated approach not only deepens fundamental knowledge of BPA degradation in biofilms but also offers practical guidance for optimizing biofilm technologies in wastewater treatment targeting endocrine-disrupting compounds.

## 2. Materials and Methods

### 2.1. Biofilm Culture and Enriched with BPA

In this study, two MBBRs (MBBR1 and MBBR2) (Yicheng, Wuxi, China) equipped with Kaldnes K3 carriers were run at different BPA concentrations. The effective working volume of each cylindrical MBBR is 4 L, and the diameter and height are 16 cm and 20 cm, respectively. The whole operation process of the reactor is divided into two stages. In the first stage, glucose is used as the only source of carbon for the formation of biofilms, which lasts for a total of 30 days. In the second stage, BPA is used as the only carbon source to enrich the biofilm. The concentration of BPA in MBBR1 and MBBR2 is 500 μg/L and 10 mg/L, and this stage lasts for 30 days. The specific operating parameters of the reactor are noted in a previous study [22], and the nutrients of the culture medium are shown in Table S1. Biofilm samples rich in BPA were used for subsequent DNA stable isotope probing experiments and DNA extraction.

### 2.2. DNA Stable Isotope Probing

Biofilm samples from two MBBRs were resuspended in the culture medium after enrichment treatments [22] for the DNA stable isotope probing (DNA-SIP) experiments. Batch experiments were conducted on the biofilm samples of each reactor, including triple 12C-BPA controls, triple 12C-BPA SIP controls, and triple 13C-BPA SIP experiments. Each experiment was carried out in a 250 mL glass container, with a liquid volume of 100 mL and an initial concentration of BPA of 10 mg/L. This concentration is higher than the typical BPA concentrations found in wastewater and used in experiments. The purpose of using such a high concentration is to ensure the availability of labeled 13C-DNA, because the biomass output of low-concentration BPA is not enough for DNA extraction and other downstream analyses. The 12C-BPA controls and 12C-BPA SIP controls contained added unlabeled BPA (99.9%, Toronto Research Chemicals, North York, ON, Canada), while 13C-BPA SIP experiments were spiked with labeled BPA (99%, Toronto Research Chemicals, North York, ON, Canada) (13C-BPA was added to the SIP experiment; for specific information, please refer to Table S2). For 12C-BPA control experiments, the BPA concentrations were measured regularly to determine the appropriate time to terminate the SIP experiments. Sludge samples were collected with a BPA removal efficiency of >90% to maximize the biodegradation and assimilation of 13C-BPA while minimizing the cross feed of 13C-BPA metabolites [14,15]. All experiments were carried out at 250 rpm at 20 ± 1 °C in a magnetic stirrer. The sludge samples collected during the experiment were temporarily stored at −20 °C for DNA extraction.

### 2.3. DNA Extraction

According to the manufacturer’s plan, the soil FastDNA Spin Kit (MP Biomedicals, Solon, OH, USA) was used to extract DNA from enriched biofilm and SIP treatment samples. The purity and concentration of the extracted DNA was determined by NanoDrop 2000 spectrophotometer (Thermo Fisher Scientific, Wilmington, DE, USA) and confirmed by agarose gel electrophoresis (1%).

### 2.4. Density Gradient Ultracentrifugation

About 5 μg DNA from each SIP treatment sample was added to Quick-Seal polypropylene tubes (13 × 51 mm, 5.1 mL; Beckman Coulter, Brea, CA, USA) and the Tris-EDTA (TE; pH 8.0)/CsCl solution was mixed to achieve a final density of about 1.72 g/mL [23]. A digital refractometer (model AR200; Leica Microsystems Inc., Buffalo Grove, IL, USA) was used to determine the density and adjusted with CsCl solution or Tris-EDTA buffer. After balancing and sealing, the tube was transferred to the ultra-fast centrifuge (Optima L-100XP; Beckman Coulter) and centrifuged at 180,000× *g* (20 °C) for 48 h [23,24]. After centrifugation, an injection pump was used to collect 10 gradient components (500 μL each) from the ultra-fast centrifuge tube. A digital refractometer was used to determine the density of each level. DNA was recovered from each grade through ethanol precipitation and resuspended in 60 μL DNA-free water.

### 2.5. 16S rRNA Gene Sequencing and Data Analysis

For the V4 high-variant region of the bacterial 16S rRNA gene and polymerase chain reaction (515F: 5′-GTG CCA GCM GCC GCG GTA-3′, 806R: 5′-GGA CTA CHV GGG TWT CTA AT-3′), the bacterial 16S rRNA gene was amplified. The total amplification capacity was 20 μL, including 4 μL 5 × FastPfu buffer, 2 μL 2.5 mM dNTPs, 0.8 μL forward primer (5 μM), 0.8 μL reverse primer (5 μM), 0.4 μL FastPfu Polymerase, and 10 ng template DNA. PCR amplification conditions were as follows: initial denaturation at 95 °C for 3 min, followed by 27 cycles of 95 °C for 30 s, 55 °C for 30 s, 72 °C for 45 s, and a final extension at 72 °C for 10 min. PCR products were purified with QIAquick PCR Purification Kit (Qiagen, Hilden, Germany) and the Qubit DNA Assay Kit and a Qubit 2.0 Fluorometer (Life Technologies, Carlsbad, CA, USA) were used for quantification. The purified PCR amplification products were used for the construction of the 16S library. Then, Agilent 2100 (Agilent Technologies Inc., Santa Clara, CA, USA) was used to analyze the 16S library pool to determine the quality and average size distribution of the library. Qubit 2.0 (Life Technologies, Carlsbad, CA, USA) was used to determine the concentration of the library pool, and then diluted to 2 nM. The qualified 16S library was sequenced on a MiSeq sequencer (Illumina, San Diego, CA, USA), and the readings of each sample exceeded 60,000.

DADA2 (version 1.26) was used to process 16S rRNA gene sequencing data to delete low-quality reading segments (sequences shorter than 150 bp in length, reading segments with a mass fraction of less than 25 phred, isopolymers greater than 6 nt, and unclear reading segments in barcodes or primers) and to merge the forward and reading segments [25]. After quality filtering, all datasets were streamlined to 54,695 sequences to achieve the same sequencing depth. The software was also used to calculate the amplicon sequence variants (ASVs), rarefaction curves, and alpha diversity index of the clean data. The ‘vegan (version 2.7-2)’ and ‘ggplott2 (version 3.5.1)’ packages in R language (version 4.2.0) were used for principal coordinate analysis (PCoA) to describe the similarity and differences between all samples’ microbial communities based on the relative abundance of ASVs.

### 2.6. Metagenomic Sequencing and Data Analysis

In order to analyze the functional genes of BPA-degrading bacteria in the biofilm, a macrogenome sequence of the biofilm samples after BPA enrichment was carried out. The DNA sequences were randomly fragmented, with an average length of about 350 bp, and were used for the preparation of the macrogenomics library. The library was diluted to 2 ng/µL, and the Agilent 2100 biological analyzer system (Agilent Technologies Inc., Santa Clara, CA, USA) was used to determine the size of the inserted fragment of the library. Then, quantitative PCR (qPCR) was used to accurately quantify the concentration of the library to ensure its quality. Then, the paired-end (2 × 150 bp) sequencing strategy was used to sequence the library on the Illumina HiSeq 4000 platform (Illumina, San Diego, CA, USA). On average, each sample generated 10 Gb of data (60 Gb in total).

Trimmomatic (version 0.39) [26] was used to process the original macrogenome readings to remove low-quality readings and trim joints. Then, the sequence was filtered to remove readings that are too short (<80 nucleotides) and readings with an average mass score of <25. By using bowtie2 (version 2.1.0) to map the readings to the human reference genome (build 37), human pollution was further eliminated (Blastn E-value threshold ≤ 10^−5^, bitscore ≥ 50, percentage identity ≥ 75%). After quality filtering, the data volume of each sample was 8.4–10.8 Gb, and the number of reads was 56.2–72.3 million. Then, MEGAHIT (version 1.2.9)was used to assemble the quality-controlled readings of each sample into overlapping groups [27]. Next, BBMap (https://sourceforge.net/projects/bbmap/ (accessed on 6 October 2023)) was used to map the quality control reading to the assembled overlap group and generate a coverage score for each overlap group. Metagenome-assembled genomes (MAGs) were then recovered from each sample using MetaBAT2 (version 2.18) [28]. The classification of each MAGs was annotated using CheckM (version 1.2.1) [29] with lineage-specific marker genes. The taxonomy of each MAG was annotated using GTDB-Tk (version 2.4.0) based on the Genome Taxonomy Database [30]. According to the annotated MAG species information, the corresponding genomes were retrieved from NCBI. FASTANI (version 1.34) [31] was used to calculate the average nucleotide identity (ANI) between the retrieved genomes and the MAGs obtained in this study. In addition, Prodigal (version 2.6.3) [32] was used to predict the functional genes in the MAGs to obtain the open reading frames (ORFs). The AromaDeg databases [33] and the KEGG database (https://www.kegg.jp/ghostkoala/ (accessed on 12 September 2023)) were used to annotate the protein sequences translated from the ORFs.

To integrate SIP and metagenomic results, we cross-referenced the taxonomic assignments of MAGs with the list of SIP-identified assimilating genera. For each matching genus, we extracted the corresponding MAGs and analyzed them for the presence of BPA degradation genes using the AromaDeg and KEGG databases as described above. The completeness and contamination of each relevant MAG were recorded to assess the reliability of gene absence calls.

### 2.7. BPA Concentration Analysis

After equipping an ultraviolet detector (DAD-3000, Agilent Technologies Inc.), high-performance liquid chromatography (HPLC) quantified the concentration of BPA in the water sample. The separation was carried out on a C18 column (2.1 × 150 mm; Agilent Technologies Inc.). The column temperature, mobile phase and flow rate, sampling volume, and detection wavelength are listed in Table S3.

## 3. Results and Discussion

### 3.1. Biodegradation of Bisphenol A

**Figure 1:**
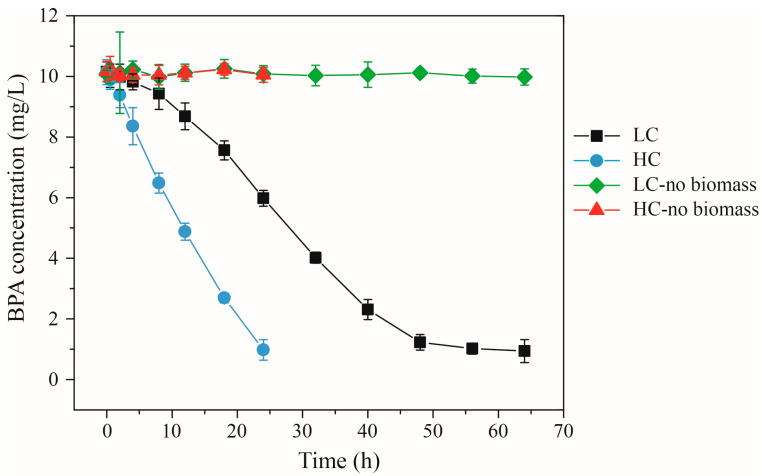
BPA concentrations for time-course evaluation reactors from SIP experiments. Results are shown for microbial communities originally derived from the MBBR bioreactors following high-concentration exposure (HC) and low-concentration exposure (LC) exposure. Results are also shown for the no-biomass controls.

Throughout the batch incubation reactors of the SIP experiment, BPA biodegradation exceeded 90% over the course of 24 and 64 h in both high-concentration (HC) and low-concentration (LC) settings, respectively (Figure 1). Each reactor exhibited an initial BPA degradation phase characterized by a lag period, succeeded by a nearly linear reduction in BPA concentration. However, it is evident that the degradation efficiencies diverged significantly across the reactors. The biodegradation coefficients, normalized to biomass, were calculated at approximately 2.83 μg/mg VSS·h for HC and around 1.36 μg/mg VSS·h for LC during this period of linear degradation. Consequently, in the context of the same exposure treatment, the microbial community within the HC biofilm exhibited swifter BPA biodegradation compared to its LC counterpart. It is a well-established fact that microorganisms act as the functional agents driving the removal of organic matter within biological treatment processes [34]. Therefore, the observed discrepancy in BPA degradation capabilities between the HC- and LC-enriched biofilms could likely be attributed to variations in microbial community structure and functionality. Additionally, under equivalent BPA enrichment concentrations, the biodegradation rate of the biofilm outperformed that of the sludge [15]. This phenomenon could potentially be attributed to dissimilarities in microbial community composition and the functional bacteria present within the two systems [35,36].

While this study controlled for pH and organic loading to isolate concentration effects, future work should examine how BOD competition, pH fluctuations, and SRT variations influence biofilm-based BPA removal in complex wastewater matrices.

### 3.2. Microbial Community Structures of BPA-Enriched Sludge

Given the potential influence of microbial community structure on function, we conducted a comprehensive analysis of the total microbial composition within each BPA-enriched sludge sample, leveraging high-throughput 16S rRNA gene sequencing. Rarefaction curves based on ASVs are shown in Figure S1: with the increase in sequences, all curves tended toward saturation, which indicates that the collected gene sequences could reasonably represent the bacterial community in biofilm at the sequencing depths used in this study. Our findings reveal distinct disparities between the bacterial communities in the high-concentration (HC) and low-concentration (LC) BPA-enriched biofilm samples (Figure S2). Notably, in LC samples, the cumulative relative abundance of Alphaproteobacteria, Betaproteobacteria, and Gammaproteobacteria exceeded 95%. Conversely, HC samples exhibited higher microbial compositional diversity, although the combined relative abundance of Alphaproteobacteria, Betaproteobacteria, Gammaproteobacteria, and Flavobacteria stood at approximately 80%. Particularly noteworthy is the significantly elevated relative abundance of Gammaproteobacteria in LC samples compared to HC samples, whereas Alphaproteobacteria and Flavobacteria prevailed in HC samples. These disparities could likely be attributed to varying BPA concentrations in the enrichment culture, given that it is widely acknowledged that nutrient concentrations wield a substantial influence over microbial community structure and the composition of functional bacteria [37] (Guo et al., 2019).

Concurrently, we undertook a comparative examination of microbial community structures within biofilms and sludge, both enriched with the same BPA concentration (10 mg/L). Within the enriched sludge, Alphaproteobacteria and Gammaproteobacteria dominated, accounting for over 80% of the relative abundance [15]. Contrastingly, the biofilm exhibited heightened microbial diversity, with a notable prevalence of Betaproteobacteria and Flavobacteria (Figure S2) [15]. The elevated microbial diversity observed in the biofilm could be attributed to several factors, including factors such as attached growth and an anaerobic environment [38].

Furthermore, we examined the relationship between microbial diversity (Shannon index) and BPA removal efficiency. Interestingly, while HC biofilms exhibited higher diversity, their degradation rates were not directly correlated with the Shannon index (ρ = 0.21, *p* > 0.05). In contrast, LC biofilms showed a moderate negative correlation between diversity and degradation rate (ρ = −0.58, *p* < 0.05), suggesting that in low-concentration systems, a less diverse but functionally specialized community may be more efficient in BPA removal.

### 3.3. Analysis of SIP Gradient Fractions

In addition to the broader composition of the microbial community, a pivotal determinant influencing the biofilm’s efficacy in BPA degradation is the presence of functional bacteria. A robust research tool, DNA-SIP, stands out as instrumental in uncovering these dynamics. To delve into this realm, we embarked on a series of batch DNA-SIP experiments centered on BPA-enriched biofilm, leveraging the power of 13C-labeled BPA as a tracer. Subsequent to the experimental phase, DNA extraction was meticulously carried out from the biofilm samples, followed by a stratification process utilizing density gradient ultracentrifugation.

**Figure 2:**
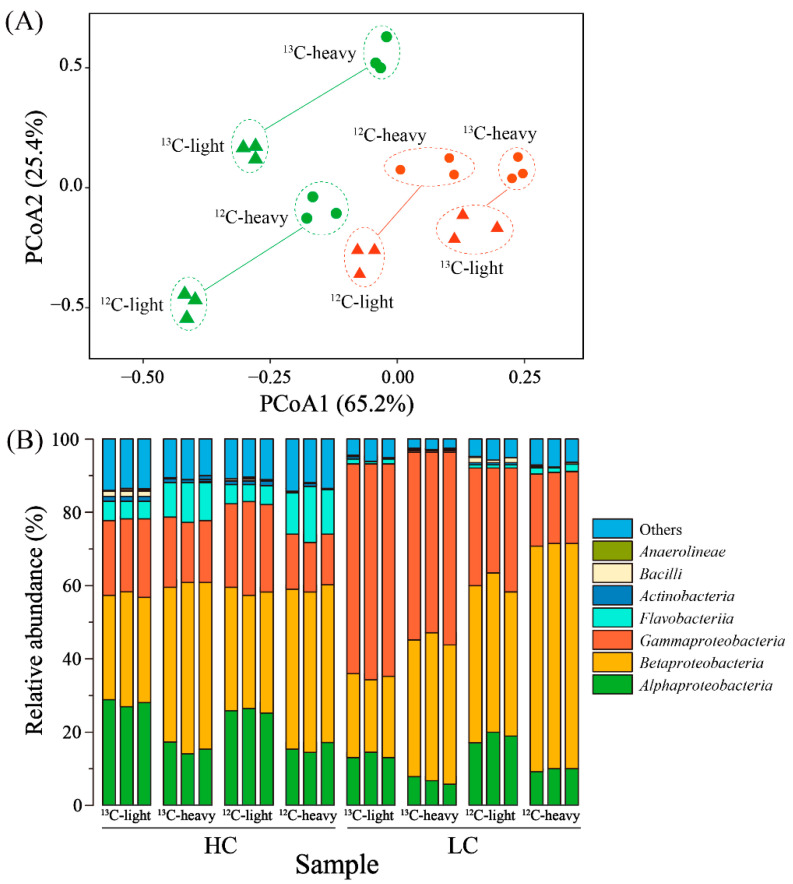
Profile of the bacterial community in upper-layer (light) and lower-layer (heavy) samples. (**A**) Principal coordinate analysis showing the bacterial community similarity among the upper and lower layers. (**B**) The microbial community compositions of the upper and lower layers at the class rank.

Following the centrifugation procedure, the density distribution across the gradient solution was meticulously profiled at various levels within the centrifuge tubes. This comprehensive data is presented in Table S4. It is evident that the density of the liquid underwent a gradual transition, diminishing from approximately 1.7 g/mL in the lower strata to around 1.5 g/mL in the upper regions. This consistent trend underscores the successful achievement of our objective: the effective isolation of 13C-labeled DNA through the density gradient ultracentrifugation technique. The discerned 13C-labeled DNA was subsequently channeled into 16S rRNA gene sequencing, which afforded a comprehensive exploration of the intricate microbial communities at play. Distinctive patterns emerged when subjecting the sequencing data to clustering analysis (Figure 2A). Most notably, this analysis yielded a clear demarcation between the upper and lower layers within each sample. This profound distinction attests to the substantial dissimilarity in microbial compositions between these layers. Importantly, this finding further bolsters the veracity of our DNA separation process, affirming the efficacy of the gradient centrifugation in successfully segregating the 13C-labeled DNA. In essence, this comprehensive methodology, encompassing DNA-SIP, 13C-labeling, density gradient ultracentrifugation, and subsequent 16S rRNA gene sequencing, has enabled a multifaceted exploration of functional bacteria’s influence on BPA degradation within biofilms. The remarkable alignment between our experimental outcomes and our initial expectations not only underscores the validity of our approach but also points towards a deeper understanding of the complex driving biofilm-mediated BPA degradation processes.

As depicted in Figure S3, the disparities in alpha-diversity indices (measured by Shannon indices) between the upper and lower layers emerged as statistically significant (as determined by a *t*-test conducted on replicated samples, with a significance level of *p* < 0.05). The computed index values for the upper layers of the high-concentration (HC) and low-concentration (LC) samples reached approximately 8 and 7, respectively. In stark contrast, the lower layers yielded markedly lower index values of approximately 4 and 3. These outcomes indicate a substantial elevation in bacterial community diversities within the upper layers of the biofilm.

The validation of these observations emerged from the microbial composition analysis, as portrayed in Figure 2B. In the context of the HC samples, the predominant microorganisms encompassed Alphaproteobacteria, Betaproteobacteria, Gammaproteobacteria, and Flavobacteria. Collectively, these taxa constituted up to 80% of the relative abundances. Notably, noteworthy disparities in composition emerged between the light (upper) and heavy (lower) layers. Specifically, Gammaproteobacteria and Flavobacteria exhibited an advantageous presence in the heavy layer. Turning attention to the LC samples, the principal microorganisms comprised Alphaproteobacteria, Betaproteobacteria, and Gammaproteobacteria, contributing to a cumulative relative abundance of approximately 90%. Here, too, marked disparities were evident between the light and heavy layers. Notably, Betaproteobacteria demonstrated prevalence in the light layer. The cumulative findings gleaned from these analyses lend further credence to the efficacy of Stable Isotope Probing (SIP) in achieving DNA stratification. This success forms the bedrock for subsequent explorations of BPA-degrading functional bacteria. By affirming the stratification of DNA based on isotopic labeling, the study underscores the reliability and viability of the analytical framework employed. This not only extends support to the broader conclusions but also serves as a cornerstone for unveiling the intricacies of BPA degradation mechanisms orchestrated by a specific microbial community.

### 3.4. Identification of Bisphenol A-Assimilating Bacteria

**Figure 3:**
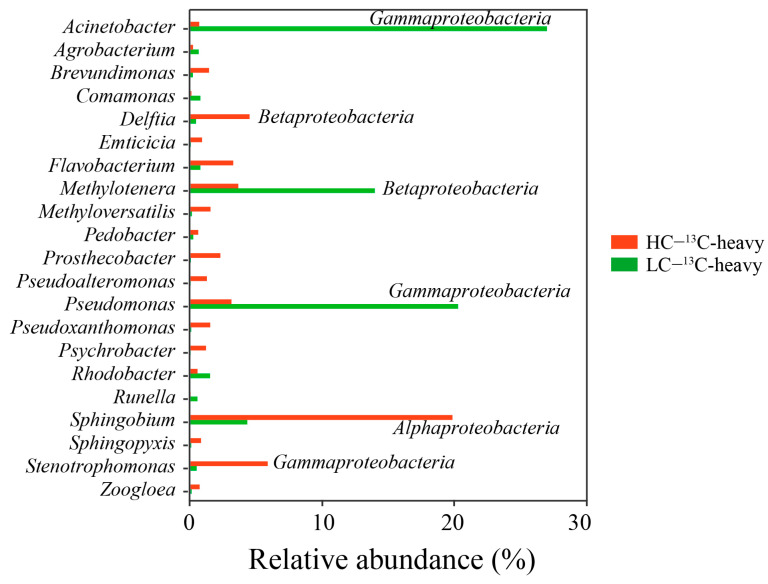
Relative abundance of genera which are present in the heavy fraction following the ultracentrifugation of DNA from the 13C-BPA SIP experiments for the HC and LC SIP experiments. Only genera present at ≥0.5% in any of the heavy fractions are shown.

Based on a comprehensive 16S rRNA gene-based phylogenetic analysis, a discernible enrichment of bacteria was observed within the 13C-heavy layer. Notably, the 13C-DNA heavy layer extracted from the LC samples exhibited distinct enrichments (27%, 13%, 20%, 4%) in bacterial taxa closely associated with *Acinetobacter*, *Methylotenera*, *Pseudomonas*, and *Sphingobium*. In contrast, the 13C-DNA heavy layer from the HC samples displayed heightened biodiversity. The primary bacterial constituents within this context encompassed *Sphingobium*, *Stenotrophomonas*, *Delftia*, *Flavobacterium*, *Methylotenera*, *Pseudomonas*, and *Prosthecobacter*, with relative abundances of 19%, 5%, 4%, 3%, 3%, 3%, and 2%, respectively (as evident from the genus-level analysis depicted in Figure 3).

**Figure 4:**
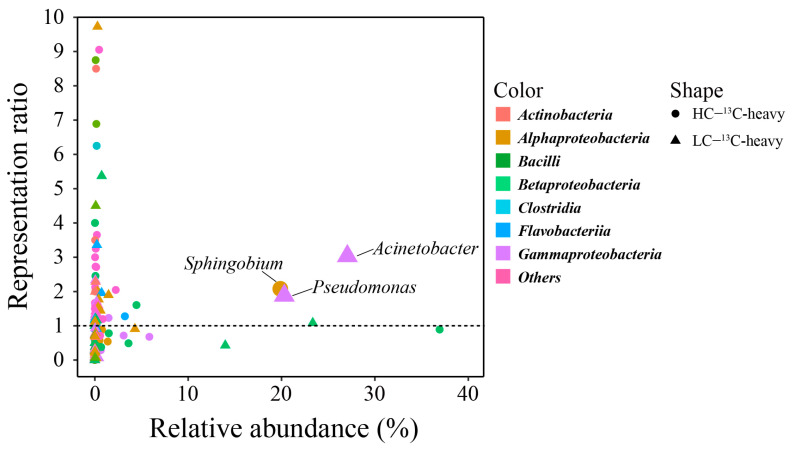
Evaluation of relative enrichment of genera based on the representation ratio and relative abundance of genera in the heavy DNA fraction from SIP experiments.

The identification of potential BPA assimilators was underpinned by their representation ratio, calculated as the relative abundance ratio of a genus in the 13C-BPA experiment’s heavy-DNA pool to the corresponding 12C-BPA control’s heavy-DNA pool. A significance threshold was established, where representation ratios exceeding one were indicative of assimilating potential. Furthermore, bacteria boasting a relative abundance surpassing 0.5% in the 13C-heavy layer were deemed the dominant assimilating bacteria. Consequently, an analysis of this nature revealed the existence of six dominant assimilating bacteria within both the HC and LC samples (as illustrated in Figure 4). In the context of HC samples, the dominant assimilating bacteria included *Delftia*, *Emticicia*, *Flavobacterium*, *Prosthecobacter*, *Pseudoxanthomonas*, and *Sphingobium*. Among these, *Sphingobium* emerged as the most notable BPA assimilating genus, constituting a relative abundance of 20%. The relative abundances of other assimilating bacteria remained below 5% (Figure S4). However, the LC samples showcased a distinct composition of dominant assimilating bacteria, encompassing *Acinetobacter*, *Agrobacterium*, *Comamonas*, *Flavobacterium*, *Pseudomonas*, and *Rhodobacter*. *Acinetobacter* and *Pseudomonas* stood out as prominent BPA-assimilating genera, contributing relative abundances of 27% and 20%, respectively (as evident in Figure S5). Further reinforcing the findings, BPA assimilating bacteria were subjected to scrutiny at the ASV level, yielding consistent outcomes. In HC samples, *Sphingobium* took center stage as the dominant assimilating genus, while in LC samples, *Acinetobacter* and *Pseudomonas* emerged as the primary players (illustrated in Figures S6–S8). Collectively, these findings underscore the diversity in dominant assimilating bacteria between HC and LC samples, with *Sphingobium*, *Acinetobacter*, and *Pseudomonas* demonstrating their pivotal roles in the BPA assimilation process across different sample contexts.

Bacteria affiliated with the genera *Sphingobium*, *Acinetobacter*, and *Pseudomonas* were isolated and their ability to thrive on BPA as the exclusive carbon source was validated in separate studies [39,40,41]. This corroborates the findings of this study, which identifies *Sphingobium*, *Acinetobacter*, and *Pseudomonas* as key BPA-assimilating bacteria. It is worth noting that the documented ability of these isolates to utilize BPA supports the accuracy of the identification process undertaken here. In conjunction with these dominant assimilators, this study unveils other bacterial species exhibiting BPA degradation capabilities. However, these candidates have yet to be isolated and cultivated, a circumstance that underscores the significance of DNA-SIP technology. This technology holds a distinct advantage given that the majority of environmental bacteria remain uncultured [42]. Although not cultivated, these bacteria show the potential for the degradation of various aromatic organic compounds. For instance, *Comamonas* showcases remarkable proficiency in the degradation of multiple aromatic organics [43] and the *Flavobacterium* genome encodes an array of functional genes dedicated to the breakdown of aromatic organic compounds [44]. The prospect of future research centered on isolating these BPA assimilating species presents an opportunity to delve deeper into the kinetics and metabolic pathways of BPA biodegradation. While this study does not directly delve into isolates, the insights gleaned through DNA-SIP lay a foundational groundwork for unraveling the degradation pathways within mixed cultures harboring newly identified assimilators. Compared with activated sludge, biofilm harbors different dominant BPA-assimilating bacteria (e.g., *Sphingobium* in high-concentration biofilm vs. *Sphingomonas* in sludge), which may contribute to the distinct degradation capabilities of the two systems. The comparison between Figures S7 and S8 reveals distinct differences in the dominant BPA-assimilating bacteria within biofilms under different BPA concentration conditions. In the high-concentration (HC) sample, *Sphingobium* emerges as the predominant assimilator, with its relative abundance substantially exceeding that of other genera. In contrast, the low-concentration (LC) sample is dominated by *Acinetobacter* and *Pseudomonas*, which collectively drive the assimilation process. Moreover, the LC sample exhibits greater microbial diversity, including other potentially degradative genera, such as *Agrobacterium* and *Comamonas*. These findings highlight BPA concentration as a key factor shaping the functional microbial community in biofilms, suggesting that the adaptability and functional specialization of distinct bacterial groups under varying concentrations may directly influence the degradation efficiency of biofilm systems.

Upon scrutinizing the BPA-assimilating bacteria within the HC and LC samples, noticeable disparities emerge. Notably, the high-concentration (10 mg/L) enrichment biofilm prominently features *Sphingobium* as the dominant assimilating bacteria, while the low-concentration (500 μg/L) enrichment biofilm showcases *Acinetobacter* and *Pseudomonas* in the role of primary assimilators. These divergent assimilating profiles could plausibly underlie the dissimilarities in biofilm BPA degradation capabilities. Nevertheless, it is imperative to acknowledge that the degradation of intricate organic substances typically involves a consortium of diverse bacteria, each contributing distinct roles within the process—a facet necessitating more comprehensive exploration [10]. Expanding the scope, it is noteworthy that significant variances extend to the BPA-dominant assimilating bacteria within both biofilm and sludge contexts, with *Sphingomonas* and *Pseudomonas* being implicated [15]. This contrast potentially underscores another contributing factor to the dissimilar degradation abilities observed. As we delve deeper into the intricacies of biofilm-mediated BPA degradation, it becomes evident that a myriad of interplaying factors influence the process. Dominant assimilating bacteria, concentration levels, and variations between biofilm and sludge microenvironments are but a few such factors. Future investigations are thus poised to illuminate the nuanced roles played by various bacterial players, as well as their dynamic interactions, ultimately elucidating the complex orchestration of BPA degradation pathways.

The differential enrichment of assimilating bacteria across concentration regimes (Figure 4) directly relates to the observed degradation kinetics, with *Sphingobium*-dominated HC communities degrading BPA 2.1 times faster than *Acinetobacter/Pseudomonas*-dominated LC communities on a biomass-normalized basis.

### 3.5. Construction of Bisphenol A Metabolic Pathway

While DNA-SIP has helped identify BPA-degrading bacteria within biofilms, the precise mechanisms underlying their degradation remain elusive. The genome of degrading bacteria achieves BPA degradation by encoding degrading enzymes [10]. We performed metagenomic sequencing on BPA-enriched biofilms. From this data, we reconstructed genomes of BPA-assimilating bacteria and identified their functional genes by cross-referencing organic degradation gene databases like KEGG and AromaDeg. Subsequently, we constructed metabolic pathways using the identified BPA-degradation functional genes.

**Figure 5:**
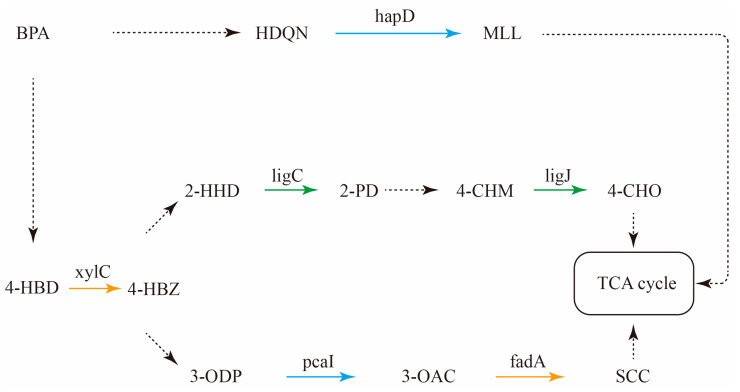
BPA metabolic pathways in the biofilm. The solid and dashed arrows represent the presence and absence of functional genes in the biofilm metagenome, respectively. Green arrows represent functional genes identified in *Sphingobium*; blue arrows represent functional genes identified in *Pseudomonas* bacteria; yellow arrows represent functional genes appearing in biofilm metagenome but not in the BPA-degrading bacteria genome analyzed by DNA-SIP. Abbreviations: BPA, bisphenol A; 4-HBD, 4-hydroxybenzaldehyde; 4-HBZ, 4-hydroxybenzoate; 2-HHD, 2-hydroxy-2-hydropyrone-4,6-dicarboxylate; 2-PD, 2-pyrone-4,6-dicarboxylate; 4-CHM, 4-carboxy-2-hydroxy-cis,cis-muconate; 4-CHO, 4-carboxy-4-hydroxy-2-oxoadipate; HDQN, 1,2,4-Benzenetriol; MLL, maleylacetate; 3-ODP, 3-oxoadipate; 3-OAC, 3-oxoadipyl-CoA; SCC, succinyl-CoA. The TCA cycle refers to the tricarboxylic acid cycle.

As depicted in Figure 5, we created three BPA metabolic pathways. However, these pathways are incomplete, with only isolated genes identified. Metagenomic sequencing alone falls short of fully capturing all functional genes in these pathways. This shortfall might stem from limitations in metagenomic sequencing technology and the degradation function gene database, a point established in previous research [10]. Combining chemical analysis with metagenomic sequencing has been the standard approach to enhance metabolic pathway accuracy [10]. Furthermore, through analysis of the host bacteria involved in the BPA metabolic pathway’s functional genes, we pinpointed genes like ligC and ligJ in the BPA-assimilating bacteria *Sphingobium*’s genome, along with hapD and pcaI genes in *Pseudomonas*’ genome. These genes are pivotal to the BPA degradation process [45], confirming the accuracy of DNA-SIP-identified BPA-assimilating bacteria to some extent. Nonetheless, we could not identify degradation function genes in other BPA-assimilating bacteria genomes. Additionally, we found degradation functional genes that were absent in BPA-assimilating bacteria genomes—genes like xylC and fadA, which are crucial for BPA degradation [45]. This might result from an inability to reconstruct the genomes of all BPA-assimilating bacteria solely through metagenomic sequencing [46]. Moreover, despite its merits as a scientific technique for identifying organic matter-degrading bacteria, DNA-SIP still falls short of completely identifying BPA-degrading bacteria [47]. It should be noted that the reconstruction of metabolic pathways based solely on metagenomic data is inherently partial due to limitations in sequencing depth, database annotation coverage, and the uncultured nature of many environmental microorganisms. In this study, the reconstructed pathways are presented as a preliminary metabolic network, with several key genes (e.g., xylC, fadA) missing from the identified genomes. This gap underscores the need for complementary approaches—such as metabolite profiling or transcriptomics—to fully elucidate the degradation pathway. Nevertheless, the identified functional genes provide a valuable foundation for understanding the cooperative degradation of BPA within biofilms.

BPA-assimilating bacteria within biofilms exhibit remarkable diversity, as illustrated in Figure 4. These bacteria possess distinct functional genes that contribute to a range of metabolic pathways. This diversity in functional genes enables these assimilating bacteria to collaborate and construct various metabolic pathways, ultimately resulting in varied biofilm degradation capabilities [10]. The BPA degradation process involves a multitude of bacterial types, with some capable of autonomously achieving BPA mineralization and removal. These bacteria exhibit notable variations in their metabolic pathways. Take, for instance, BPA-assimilating bacteria like *Sphingomonas*, *Pusillimonas*, and *Pseudomonas*, each of which follows a distinct metabolic pathway [10].

In environments characterized by high biodiversity, relying solely on a single bacterial type to degrade organic matter might not be the most common or optimal approach. Typically, effective mineralization and the removal of specific organic compounds involve the cooperation of multiple bacterial species; this cooperative principle holds true for the degradation of BPA [10]. Referencing Figure 5, it becomes apparent that the functional genes present in each assimilating bacterium contribute to only a fraction of the complete degradation pathway. For example, the reactants necessary for the metabolic steps governed by the ligC gene arise from the products of metabolic steps controlled by the xylC gene. Intriguingly, these two crucial genes are not found within the same bacterial genome. Thus, comprehending the intricate interplay among diverse assimilating bacteria in the BPA degradation process becomes pivotal for a comprehensive grasp of BPA biofilm degradation dynamics. Ultimately, acknowledging the intricate relationships and collaborations among different assimilating bacteria facilitates a deeper understanding of the fundamental mechanisms driving BPA degradation within biofilms.

## 4. Conclusions

The predominant assimilating bacteria in biofilms enriched with high and low concentrations of BPA differ, and these populations also contrast with those found in activated sludge.

The degradation function genes within distinct BPA-assimilating bacteria exhibit substantial variations, and these genes play a pivotal role in shaping diverse metabolic pathways.

## Data Availability

The datasets are available from the corresponding author upon reasonable request.

## Supplementary Materials

The following supporting information can be downloaded at: https://www.mdpi.com/article/10.3390/toxics14010080/s1, Figure S1: Rarefaction curves based on sequencing; Figure S2: The microbial community compositions of BPA-enriched sludge samples at Class level; Figure S3: Shannon indices of the light- and heavy-layer samples; Figure S4: Relative abundance of BPA-dominant assimilating bacteria (genus level) in HC samples; Figure S5: Relative abundance of BPA-dominant assimilating bacteria (genus level) in LC samples; Figure S6: Evaluation of relative enrichment of ASV based on the representation ratio and relative abundance of ASV in the heavy DNA fraction from SIP experiments; Figure S7: Relative abundance of BPA-dominant assimilating bacteria (ASV level) in HC samples; Figure S8: Relative abundance of BPA-dominant assimilating bacteria (ASV level) in LC samples; Table S1: Components of the medium used in the biofilm culture experiments; Table S2: Basic information of 13C-BPA; Table S3: Parameters of HPLC analysis used to measure BPA concentrations; Table S4: Density of the liquid at different heights obtained from ultracentrifugation; Table S5: MAGs classification and completeness information.
