# Supplementary material for: Integrated 13C-DNA Stable Isotope Probing and Metagenomics Approaches to Identify Bisphenol A Assimilating Microorganisms and Metabolic Pathways in Biofilms
Source: Toxics. 2026 Jan 15;14(1):80. doi: 10.3390/toxics14010080 (PMC12846180; doi:10.3390/toxics14010080)
Supplement: Supplementary file 1 [file toxics-14-00080-s001.zip › toxics-4049639-supplementary.pdf]

Supplementary Material

Integrated 13C-DNA Stable Isotope Probing and Metagenomics Approaches to Identify Bisphenol A Assimilating Microorganisms and Metabolic Pathways in Biofilms

Di Wang [1], Jiayue Sun [1], Yunian Zhang [1], Lingjue Yuan [1], Xia Xu [2], Yingang Xue [1,*] and Haohao Sun [1,*]

[1] School of Environmental Science and Engineering, Changzhou University, Changzhou 213164, China; wangdidi0625@163.com (D.W.); s23030857049@smail.cczu.edu.cn (J.S.); zhangyunian111@163.com (Y.Z.); 15352171818@163.com (L.Y.)
[2] College of Urban Construction, Changzhou University, Changzhou 213164, China; xuxia@cczu.edu.cn
* Correspondence: xyg@cczu.edu.cn (Y.X.); shh@cczu.edu.cn (H.S.)

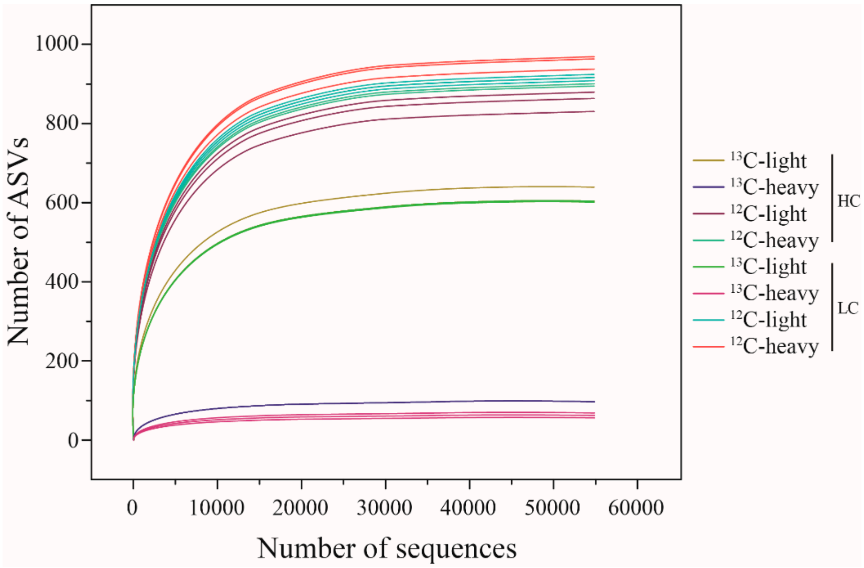

Figure S1. Rarefaction curves based on sequencing.

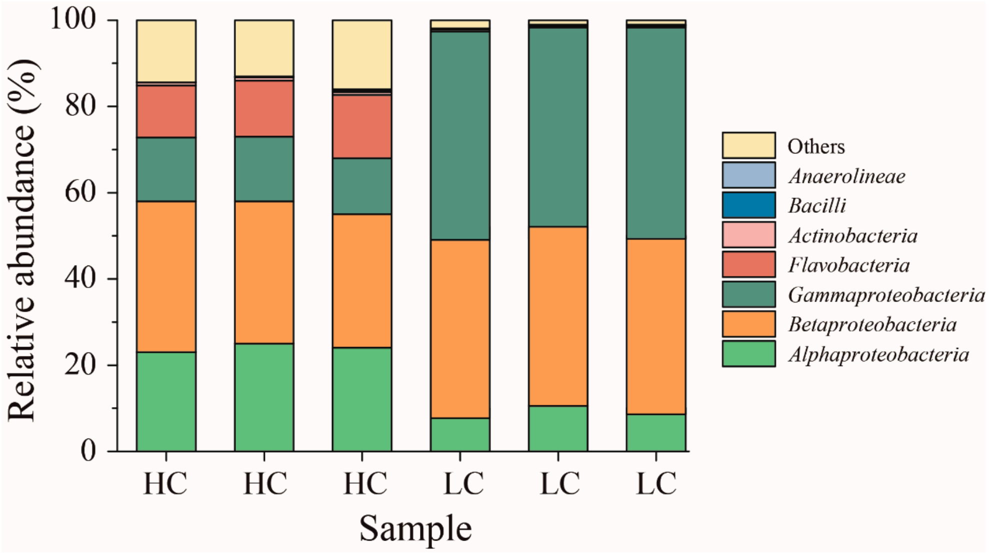

Figure S2. The microbial community compositions of BPA-enriched sludge samples at Class level

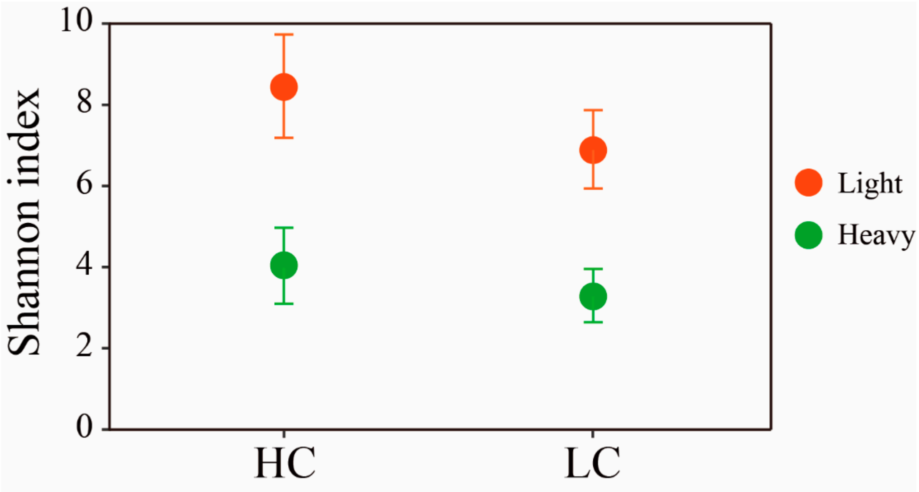

Figure S3. Shannon indices of the light- and heavy-layer samples

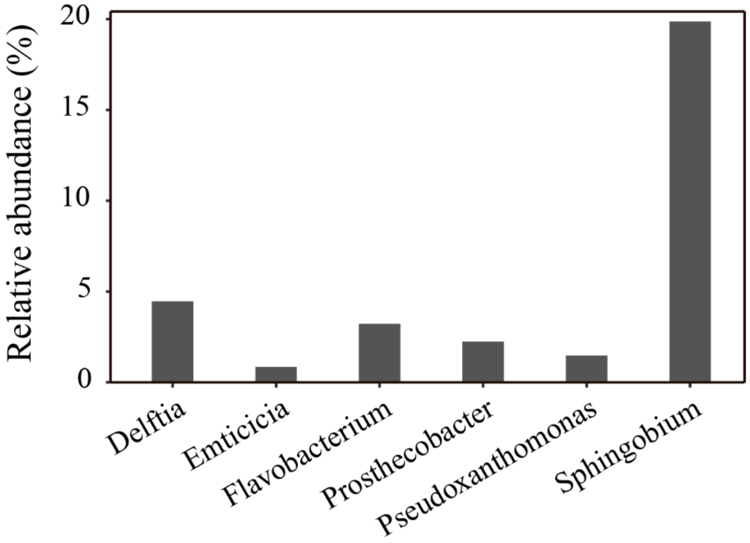

Figure S4. Relative abundance of BPA-dominant assimilating bacteria (genus level) in HC samples

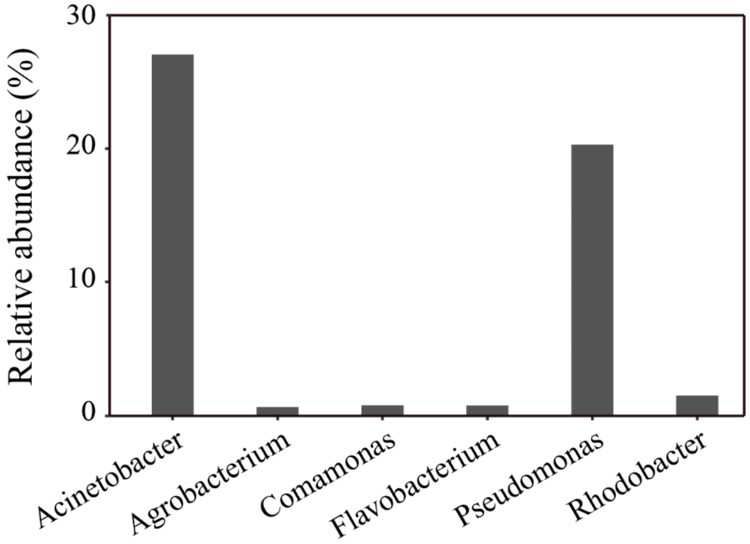

Figure S5. Relative abundance of BPA-dominant assimilating bacteria (genus level) in LC samples

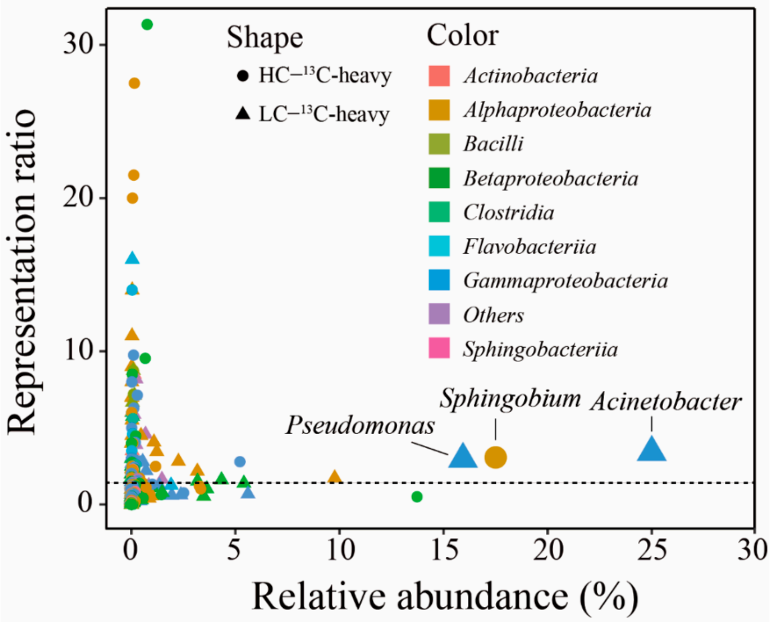

Figure S6. Evaluation of relative enrichment of ASV based on the representation ratio and relative abundance of ASV in the heavy DNA fraction from SIP experiments.

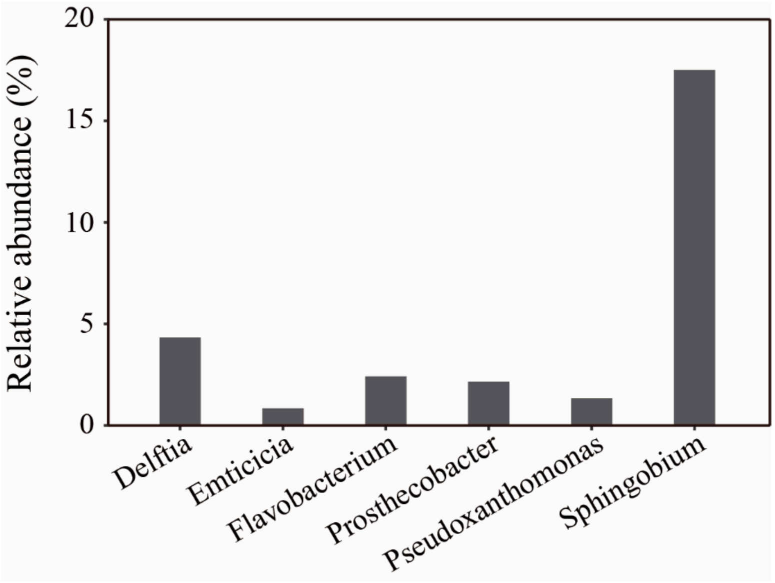

Figure S7. Relative abundance of BPA-dominant assimilating bacteria (ASV level) in HC samples

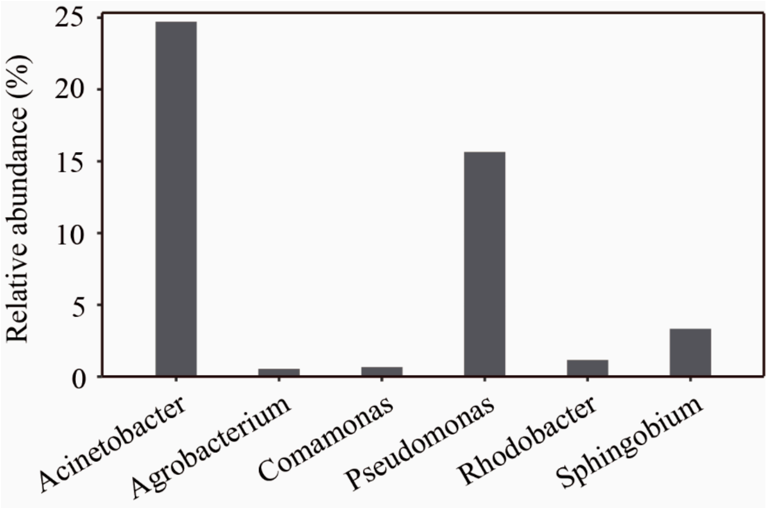

Figure S8. Relative abundance of BPA-dominant assimilating bacteria (ASV level) in LC samples

Table S1. Components of the medium used in the biofilm culture experiments.

**Component**	**Concentration (mg/L)**
$NaHCO_3$	188
$K_2HPO_4 \cdot 3H_2O$	24
$FeSO_4 \cdot 7H_2O$	22
KH_2PO_4	14
$CaCl_2 \cdot 2H_2O$	7.5
$MgSO_4 \cdot 7H_2O$	3.5
$NiSO_4 \cdot 6H_2O$	0.7
$MnSO_4 \cdot H_2O$	0.25
$ZnSO_4 \cdot 7H_2O$	0.05
$(NH_4)_6Mo_7O_{24} \cdot 4H_2O$	0.01
$CuCl_2 \cdot 2H_2O$	0.01
$Co(NO_3)_2 \cdot 6H_2O$	0.01

Table S2. Basic information of ^{13}C-BPA

Name	Abbreviation	Formula	CAS	Chemical Structure
Bisphenol A	BPA	$C_{15}H_{16}O_2$	80-05-7	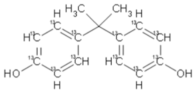

Table S3. Parameters of HPLC analysis used to measure BPA concentrations

Chromatographic column	Column temperature (℃)	Detection wavelength (nm)	Flow velocity (mL/min)	Mobile phase (v/v)	sample size (uL)
C_{18}	Room temperature	275	1	0.1% acetic acid:methanol=60:40	20

Table S4. Density of the liquid at different heights obtained from ultracentrifugation

**Sample**	**Density (g/mL)**									
	**Layer1 (top)**	**Layer2**	**Layer3**	**Layer4**	**Layer5**	**Layer6**	**Layer7**	**Layer8**	**Layer9**	**Layer10 (bottom)**
HC-^{13}C-1	1.619	1.631	1.653	1.657	1.668	1.686	1.712	1.734	1.755	1.777
HC-^{13}C _2	1.613	1.624	1.635	1.651	1.671	1.705	1.724	1.743	1.769	1.774
HC-^{13}C _3	1.552	1.577	1.635	1.646	1.668	1.673	1.687	1.713	1.739	1.748
HC-^{12}C-1	1.613	1.618	1.628	1.645	1.662	1.679	1.704	1.724	1.743	1.764
HC-^{12}C _2	1.584	1.608	1.620	1.636	1.657	1.681	1.717	1.714	1.749	1.768
HC-^{12}C _3	1.582	1.596	1.601	1.628	1.654	1.693	1.703	1.726	1.743	1.761
LC-^{13}C-1	1.573	1.581	1.618	1.651	1.675	1.682	1.693	1.726	1.748	1.759
LC-^{13}C _2	1.577	1.602	1.633	1.646	1.663	1.694	1.715	1.733	1.754	1.772
LC-^{13}C _3	1.598	1.616	1.622	1.641	1.662	1.703	1.721	1.723	1.752	1.763
LC-^{12}C-1	1.586	1.604	1.626	1.640	1.678	1.691	1.712	1.731	1.748	1.760
LC-^{12}C _2	1.627	1.652	1.663	1.673	1.686	1.703	1.721	1.734	1.752	1.761
LC-^{12}C _3	1.651	1.657	1.661	1.678	1.684	1.696	1.716	1.728	1.748	1.759

Table S5. MAGs classification and completeness information

MAGs	Classify	Completenes
Bin2	Sphingobium	96.23
Bin5	Prosthecobacter	85.3
Bin7	Prosthecobacter	90.12
Bin12	Flavobacterium	88.88
Bin14	Sphingobium	95.12
Bin17	Pseudomonas	98.37
Bin19	Flavobacterium	91.48
Bin20	Pseudomonas	98.90
Bin23	Sphingobium	98.13
Bin35	Flavobacterium	80.08
